# A nationwide survey of patient problem occurrence across different nursing healthcare sectors

**DOI:** 10.1002/nop2.102

**Published:** 2017-10-12

**Authors:** Renate Kieft, Anke de Veer, Anneke Francke, Diana Delnoij

**Affiliations:** ^1^ Dutch Nurses’ Association Utrecht The Netherlands; ^2^ Netherlands Institute for Health Services Research (NIVEL) Utrecht The Netherlands; ^3^ Department of Public and Occupational Health EMGO+/VU Medical Center Amsterdam The Netherlands; ^4^ TS Social and Behavioral Sciences Tranzo Scientific center for care and welfare Tilburg University Tilburg The Netherlands

**Keywords:** influence, nurses, patient problems, survey

## Abstract

**Aim:**

The aim of this study was to determine the patient problems that nurses encounter in different clinical settings and the extent to which they report being able to influence those patient problems.

**Design:**

Exploratory online survey research.

**Method:**

Data were collected through an online questionnaire. We prepared a 2 × 2 matrix to compare the rate of occurrence against the average level of reported influence. Descriptive statistics were used for the data analysis.

**Results:**

A total of 440 nurses working in different settings completed the questionnaire. Nurses report having the most influence on patient problems related to self‐care, mobility and functions of the skin. Nurses experience less influence on problems with voice/speech and the tasks required for participation in work/employment.

## 1 INTRODUCTION

Nurses provide care to people of all ages in various healthcare settings such as hospitals, residential care, general practices, primary care, psychiatric health care and care for the disabled. Nurses with various levels of education work together in collaboration with other healthcare professionals (Jacob, Mckenna, & D'Amore, 2015). The focus of nursing care can differ between clinical settings. For instance, psychiatric health nurses take care of patients with mental and emotional disorders (eg, depression, schizophrenia) and focus on coping and adjustment of anxiety or mood problems (MacNeela, Morris, Scott, Treacy, & Hyde, 2010). Hospital nursing care might be more concentrated on patients with physical diseases, such as heart failure or cancer and nursing care could be focused on the coping and adjustment of pain, dyspnoea or nausea (Griffiths, Richardson, & Blackwell, 2012). Although the focus of nursing care can differ between clinical settings, the problems or health issues that patients experience are not restricted to one specific setting. For instance, a patient with severe mobility problems has an increased risk of developing pressure ulcers, regardless of the healthcare setting where the patient resides. From the patient's perspective, it is important that nursing care can be continued and that nursing information is up‐to‐date, accurate and not contradictory. From the perspective of nurses, it is important to have an actual record of the nursing care process that a patient has gone through and which can follow the patient after transfer to another setting.

The information nurses gather, share and exchange should therefore be used or reused when a patient is transferred from one setting to another. However, a retrospective patient record review showed variation in what nurses write in patient records in Dutch hospitals. Patient problem labels (*N* = 1635) with variances in descriptions were ascertained in 369 nursing records (Paans & Müller‐Staub, 2015). Similarly, other studies on the transfer of information also found a wide variability of information in the nursing records (Griffiths, Morphet, Innes, Crawford, & Williams, 2014; Holly & Poletick, 2014). The variation and variability hampers the exchange and reuse of data within and across settings (Hughes, Lloyd, & Clark, 2008; Lavin, Harper, & Barr, 2015; Voyer, Cole, McCusker, St‐Jacques, & Laplante, 2008). It is therefore essential to have a clear view of patient problems that commonly occur in clinical nursing practice across different healthcare settings.

### 1.1 Background

Patient problems form the basis for a nursing care plan where nurses make clinical decisions in agreement with the patient and/or their close relatives, coordinate care, set goals and monitor care results (Johansen & O'Brien, 2016). Throughout this paper, the term “patient problem” will be used as a synonym for a nursing diagnosis, health or health‐related issues, phenomena or problems. One essential aspect of identifying a patient problem is that nurses can plan interventions and actions to help the patient to achieve positive results (Lavin et al., 2004). For example, when an area of skin is placed under pressure, with appropriate interventions nurses can prevent that pressure ulcer emerges. In general, the scope of nursing care is focused on patient problems arising from an illness, disorder or disability and contributes to maintaining or restoring health, the ability to function and quality of life. The illness itself is not necessarily the focal point; rather, that is how the patient functions. This is viewed as an interaction between the illness or disorder on the one hand and, on the other, the ability to function and participate in a social context (Royal College of Nursing (RCN), 2014). Patient problems defined by nurses should therefore reflect and capture this scope.

On the other hand, there is a different perception about the inclusion of patient problems related to nursing practice. For instance, the classification of nursing diagnosis as developed by Nanda‐International included a nursing diagnosis of “feeding self‐care deficit” (Nursing Diagnoses 2015‐2017: Definitions and Classification, 2014), which is not included as a problem by the Omaha System classification (Koster & Harmsen, 2015). Besides, nurses also describe patient problems in their own words (Paans & Müller‐Staub, 2015), leading towards a diversity of patient problems and definitions. It could be argued that nurses do not have access to consistent and coherent nursing information, including patient problems. To determine which patient problems reflect and capture the scope of the nursing clinical practice, identifying the occurrence of relevant patient problems is a necessary first step (Coenen & Kim, 2010).

The aim of this research was to gain more insights into the occurrence of patient problems in the Dutch clinical nursing practice. In the Netherlands, running a query to identify which patient problems occur in nursing practice is difficult, because nursing care is mostly reported by hand in patient records (as narrative text). We therefore conducted a survey study among Dutch nurses across different healthcare settings to determine what patient problems they encounter. We also examined the extent to which they report being able to influence (prevent or minimise) patient problems. The extent of the influence that nurses experience in preventing or minimising patient problems may give an insight into which patient problems are relevant to nursing care (Heslop & Lu, 2014). This present study has been set up to gain more insight in the type of patient problems needs to be shared in the context of the clinical nursing practice across different healthcare setting and populations.

#### 1.1.1 Research questions

1. Which categories of patient problems do nurses encounter in clinical practice most frequently?
2. Which specific patient problems do nurses encounter daily?
3. What level of influence do nurses report having in preventing or minimising patient problems that occur daily?

## 2 METHOD

### 2.1 Research design

Exploratory online survey research.

### 2.2 Sample and recruitment process

For this study, 838 registered nurses were approached who had expressed willingness to complete online questionnaires. These nurses were participants in a pre‐existing survey panel, the Nursing Staff Panel (http://www.nivel.nl/panelvenv). The Nursing Staff Panel was recruited through a previous survey among a representative random sample of Dutch healthcare employees working in the largest healthcare sectors in the Netherlands (ie, hospitals, mental health care, general medical practice, home care, healthcare for the disabled and residential care for the elderly) and who were known and had been approached by the Dutch Employee Insurance Agency (UWV). This agency is responsible for social security payments and records all employees in the Dutch healthcare sector. Only nursing staff providing direct patient care was invited to become participants of the Nursing Staff Panel. This procedure encouraged a diverse and representative composition for the panel in terms of age, gender, region and employer (de Veer, Francke, Struijs, & Willems, 2013; Kroezen, de Veer, Francke, Groenewegen, & van Dijk, 2014).

### 2.3 Developing the online questionnaire

**Figure 1:**
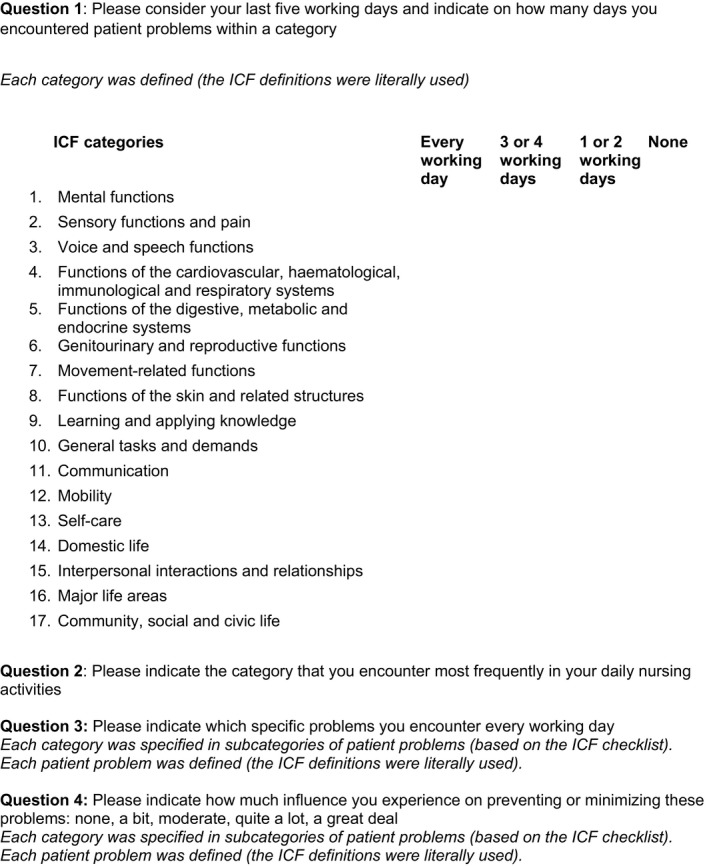
The online questionnaire

As the aim of this study was to gain more insight into the occurrence of patient problems across different healthcare sectors, a questionnaire was set up (Fig. 1). The questionnaire was based on the theoretical framework of the International Classification of Functioning, Disability and Health (ICF), because of its conceptualization of health and health‐related functioning (RIVM, 2007). Nurses examine the relationships between disorders, limitations in activity and functioning and care for patients in different healthcare contexts (Heinen, van Achterberg, Roodbol, & Frederiks, 2005; Kearney & Pryor, 2004). The ICF approaches human functioning from three perspectives: the body, the individual and the social aspects (RIVM, 2007). The human organism is classified into organ systems, identified as the “body functions and structure” component. The second and third perspectives are addressed using the “activity and participation” component. Both components, “body functions and structure” and “activity and participation”, are divided into 17 categories. These categories are in turn subdivided into subcategories with terms and descriptions. A category can include several subcategories. To address all aspects of patient problems from the different healthcare contexts, the patient problems were systematically organised by using the sorting of the ICF checklist (World Health Organization, 2003). The researcher checked if the categories could be connected to nursing practice and added a subcategory if necessary. Each category and subcategory was defined. The ICF definitions were literally incorporated into the online questionnaire (http://apps.who.int/classifications/icfbrowser/). The final categories and subcategories are shown in Appendix 1.

### 2.4 The Questionnaire

For Question 1, the respondent was shown the 17 categories and asked to state the number of days during the preceding period of five working days on which they encountered patient problems (see Fig. 1, Question 1). An explanation accompanying the question stated that it was irrelevant whether the problem occurred repeatedly with the same patient or with various patients.

Categories marked by respondents as “every working day” were counted automatically by the survey software. If a respondent gave this answer in more than seven categories, they were asked a supplementary question (Question 2). All respondents were subsequently shown the categories they had indicated (up to a maximum of seven) and asked to state which specific problems they encounter every working day (Question 3). An explanation accompanying the question, where each patient problem was defined in accordance with the definitions of the Dutch translation of the ICF (RIVM, 2007). The respondents were next asked to indicate how much influence they have in preventing or minimising problems (Fig. 1, Question 4), with five possible answers: “none” (score 1), “a bit” (score 2), “moderate” (score 3), “quite a lot” (score 4) and “a great deal” (score 5).

To test the content validity of the draft questionnaire, a researcher (RK) approached seven experts (known by the researcher). The experts had a background in nursing and were familiar with the ICF. The experts had no suggestions. Fifteen professionals with backgrounds in nursing tested the face validity of the questionnaire. The professionals were recruited by the board members of the departments of the Dutch Nurses’ Association (http://www.venvn.nl/Afdelingen). The professionals recruited were approached by email. Their comments concerned textual adjustments, which were literally incorporated into the drafted questionnaire.

### 2.5 Data collection

Subsequent to the test phase, an e‐mail containing a hyperlink to the questionnaire was sent to 838 nurses. These nurses were participants in the Nursing Staff Panel (http://www.nivel.nl/panelvenv). The e‐mail explained the objective and importance of the research. The respondents could complete the questionnaire anonymously. Nurses who had not yet done so were sent a maximum of three e‐mail reminders at intervals of 2 weeks.

### 2.6 Ethical considerations

All respondents received a letter explaining the objective of the study and stating that participation was voluntary. Further ethical approval of this study was not required under the legislation (www.ccmo.nl/en/) applicable in the Netherlands, as all respondents were competent individuals and this study did not involve any interventions or treatments.

### 2.7 Data analysis

The data collected were exported to SPSS (versions 18 and 21). The frequencies of specific categories were arranged according to rate of occurrence and collated in a table. Next, the frequencies of the patient problems in each specific category were computed and sorted in descending order from most to least. Two groups were created by using the median to identify the 50% most frequently occurring and 50% least frequently occurring patient problems. The median frequency was 65.5 with a minimum of 4 and a maximum of 185. Similarly, we used the median to form two groups of level of influence: “high level” and a “low level” of perceived influence. The median level was 2.96 with a minimum of 1.83 and a maximum of 3.68. A 2 × 2 table was then used to combine the frequency of occurrence with the level of reported influence. This created four quadrants: (i) frequently occurring/high level of influence experienced, (ii) frequently occurring/low level of influence experienced (iii) less frequently occurring/high level of influence experienced and (iv) less frequently occurring/low level of influence experienced. The four quadrants provide a framework by which patient problems and the level of reported influence can be explored and analysed further.

## 3 RESULTS

In February and March 2014, 440 of the nurses approached completed the questionnaire (response rate of 52.5%). Of these, 377 (86%) were female (see Table 1). The average age of the respondents was 49 (standard deviation, or *SD* 10.2). The majority have a Bachelor's degree in nursing (53%), while 35% have an Associate degree and 2% a Master's degree. The largest group are those employed at hospitals (35%), followed by psychiatric healthcare (17%), general medical practice (16%), primary care (15%), health care for the disabled (11%) and residential care for the elderly (6%).

**Table 1:** Demographics (*N *=* *440)

Demographics	Mean (%) *SD*
Gender	
Female	377 (86%)
Male	63 (14%)
Age	49 (24‐64) *SD* 10,2
Education level	
Nurses with an Associate degree	156 (35%)
Nurses with a Bachelor's degree	233 (53%)
Nurses with a Master's degree	10 (2%)
Unknown	41 (9%)
Health care sector	
Hospital care	155 (35%)
Psychiatric health care	73 (17%)
General medical practice	72 (16%)
Primary care	65 (15%)
Disability health care	48 (11%)
Residential elderly care	27 (6%)
Work experience in years, mean (range)	24 (1–46) *SD* 10,6
Working hours per week, mean (range)	28 (5–40) *SD* 6,9

### 3.1 Most commonly occurring categories of patient problems

**Figure 2:**
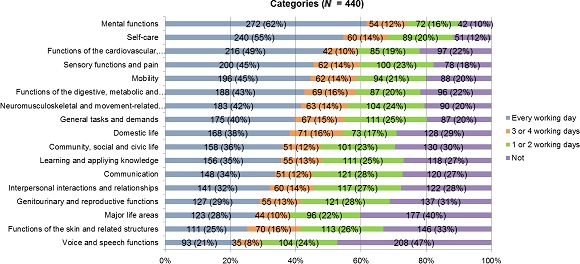
Categories of patient problems in the health care sector as a whole

A total of 88% of respondents reported encountering one or more categories of patient problems “every working day”. Figure 2 shows that 62% of respondents encounter patient problems in the category “mental functions” on a daily basis, followed by the categories “self‐care” (55%) and “functions of the cardiovascular, haematological, immunological and respiratory systems” (49%). The least reported categories were “voice and speech functions” (21%), “functions of the skin and related structures” (25%) and “major life areas” (28%).

### 3.2 Specific patient problems and the level of influence reported

Table 2 displays the results according to the rate of occurrence and the average reported level of influence. The “Cat.” column indicates the category containing the specific patient problem. Column “*n*” states the number, that is, how often a patient problem was encountered on a daily basis. The “Mean i” column gives the average level of influence that respondents reported.

**Table 2:** Patient problems compared to level of reported influence

**Quadrant 1**		***n***	**Mean I**	**Quadrant 2**		***n***	**Mean I**
**Cat**	**Frequently occurring/high level of influence experienced**			**Cat**	**Frequently occurring/low level of influence experienced**		
5	Defecation	87	3.64	15	Complex interpersonal interactions. such as forming or terminating relationships	81	2.96
13	Washing oneself	185	3.64	7	Functions of the joints and bones	120	2.95
13	Dressing	164	3.64	4	Heart functions. including heart rate. rhythm	130	2.94
13	Toileting	151	3.61	1	Energy and drive functions	76	2.92
2	Pain and sensation of pain	107	3.54	1	Attention	147	2.91
13	Caring for body parts	165	3.54	1	Temperament and personality functions	113	2.90
13	Eating and drinking	97	3.51	1	Orientation	137	2.88
5	Water. mineral and electrolyte balance functions	81	3.50	1	Perceptual functions	69	2.86
12	Changing and maintaining body position	116	3.45	4	Blood vessel function	106	2.80
4	Blood pressure functions	131	3.44	17	Community life	77	2.80
4	Respiratory system	104	3.41	1	Experience of self and time functions	82	2.74
5	Weight maintenance	92	3.39	1	Thought functions	127	2.60
10	Carrying out daily routine	81	3.38	7	Muscle power functions	79	2.57
10	Undertaking a single or multiple tasks	81	3.29	1	Memory	138	2.53
13	Looking after one's health	164	3.28	1	Intellectual functions	114	2.25
9	Solving problems	77	3.27	**Cat**	**Quadrant 4**	***n***	**Mean I**
					**Less frequently occurring/low level of influence experienced**		
12	Moving around using transportation	76	3.22	15	Particular interpersonal interactions. such as relating with strangers. formal relationships. family and intimate relationships	68	2.95
1	Emotional functions	167	3.21	11	Conversation	61	2.93
10	Handling stress and other psychological demands	89	3.18	5	Endocrine gland functions	30	2.85
12	Carrying. moving and handling objects	79	3.18	6	Sensations associated with urinary functions	26	2.84
11	Communicating ‐ receiving	88	3.10	6	Urinary excretory functions	42	2.80
12	Walking and moving	135	3.08	9	Sensory experiences	16	2.80
11	Communicating ‐ producing	74	3.07	6	Urination functions	54	2.77
17	Recreation and leisure	72	3.06	1	Consciousness	61	2.75
14	Household tasks	97	3.02	4	Functions of the immunological system	41	2.62
15	Basic interpersonal interactions	82	3,00	17	Religion and spirituality	20	2.60
1	Sleep	147	2.99	16	Work and employment	38	2.58
**Quadrant 3**		***n***	**Mean I**	6	Sexual functions	9	2.56
**Cat**	**Less frequently occurring/high level of influence experienced**						
8	Protective functions of the skin	44	3.68	7	Sensations related to muscles and movement functions	63	2.56
4	Sensations associated with cardiovascular and respiratory functions	52	3.50	16	Education	24	2.55
5	Thermoregulatory functions	43	3.46	14	Acquiring a place to live	29	2.52
6	Sensations associated with genital and reproductive functions	5	3.40	16	Economic life	43	2.49
8	Functions of the hair and nails	14	3.38	2	Hearing	60	2.44
8	Repair functions of the skin	28	3.33	7	Muscle endurance functions	21	2.42
5	Ingestion functions	49	3.29	6	Menstruation functions	5	2.40
5	Functions related to metabolism system	58	3.23	9	Basic learning and applying knowledge	37	2.39
11	Communication devices and techniques	13	3.18	7	Muscle tone functions	51	2.36
5	Sensations associated with the digestive system. including nausea. feeling bloated etc.	56	3.16	2	Taste. smell and touch function	43	2.30
8	Sensation related to the skin	23	3.14	6	Procreation functions	4	2.25
5	Digestive functions	28	3.04	7	Involuntary movement functions	31	2.20
14	Shopping and gathering daily necessities	65	3.03	2	Seeing	45	2.17
4	Functions of the haematological system	58	3,00	3	Voice function	20	1.95
				3	Fluency and rhythm of speech functions	18	1.94
				3	Articulation	31	1.83

Quadrant 1 (frequently occurring/high level of influence experienced) and quadrant 3 (less frequently occurring/high level of influence experienced) contain patient problems that respondents said they had a high level of influence over in terms of prevention or minimization. Problems related to the “functions of the skin and related structures” (category 8), “general tasks and demands” (category 10), “mobility” (category 12) and “self‐care”(category 13) are particularly striking. Nurses reported having a high level of influence over all the problems in these categories, irrespective of the rate of occurrence.

Quadrant 2 (frequently occurring/low level of influence experienced) and quadrant 4 (less frequently occurring/low level of influence experienced) contain patient problems that respondents said they had a low level of influence over. In this case, all the problems related to “voice and speech functions” (category 3), “neuromusculoskeletal and movement related functions” (category 7) and “major life areas” (category 16) are particularly striking. Irrespective of the rate of occurrence, respondents stated they had a low level of influence when it came to preventing or minimising problems in these categories. Nurses also experience a low level of influence over most of the problems in the category “mental functions” (category 1), except over problems with “emotional functions” and “sleep”. The latter two are included in quadrant 1 (frequently occurring/high level of influence).

## 4 DISCUSSION

Using an online survey, we collected information about patient problems in the clinical nursing practice across different healthcare settings and the level of influence nurses say they have in preventing or minimizing these problems. The first research question aimed to gain more insight into the occurrence of categories of patient problems. Our study showed that mental functions, self‐care and the functions involved in the cardiovascular system, haematological, immunological systems and the respiratory system were frequently occurring categories. An interesting finding is that a category can have a high rate of occurrence, but nurses do not necessarily perceive any influence on all patient problems included in the specific category. For instance, the category “cardiovascular system, haematological, immunological systems and the respiratory system” was ranked as a frequently occurring. Looking at the specific patient problems included, nurses experienced a high level of influence on a less frequently occurring patient problem related to “sensations associated with cardiovascular and respiratory functions” (quadrant 3) in contrast to the patient problem “heart functions, including heart rate, rhythm” (quadrant 2: frequently occurring/low level of influence).

When we consider the “high level of influence” more closely, we found that nurses feel they are in a position to influence a considerable number of patient problems (quadrants 1 and 3); related to washing, dressing, eating/drinking, pain, respiratory functions and handling stress. When reviewing the results, we found that our findings are broadly consistent with several studies. Doran's extended analysis of the evidence to include nursing outcomes in acute, community, home and long‐term healthcare settings (Doran, 2011) confirmed that patient problems related to pain, symptom management (including fatigue, nausea and vomiting), dyspnoea and adverse patient outcomes (including pressure ulcers) can be affected by nursing care. Also, functional status (containing washing and drying yourself, dressing, toileting, eating, household activities and getting from bed to chair) as well as psychological distress are seen as nursing‐sensitive, along with emotional functioning, handling stress and sleeping problems. Escalada‐Hernández et al. (Escalada‐Hernández et al., 2015) performed a retrospective study that identified the nursing diagnoses of 690 patients with psychiatric illnesses. They found that common nursing diagnoses related to self‐care deficits, including bathing, dressing, feeding, ineffective health management. The study by Paans & Müller‐Staub (Paans & Müller‐Staub, 2015) conducted in ten hospitals found the most prevalent patient problems to be acute pain, nausea, fatigue, feeding and risk of impaired skin integrity.

When we consider the “low level of influence” more closely, we found that nurses feel they have a low level of influence (quadrant 2 and 4) on several patient problems, eg, patient problems with attention, perception, memory, thought, orientation or problems associated with hearing, speaking, voice, urination, religion, work/economic life. In reviewing the results, we found that both the study by MacNeela et al. (MacNeela et al., 2010) on the scope of mental health nurses and the study by Escalada‐Hernández et al. (Escalada‐Hernández et al., 2015) found prevalent patient problems related to thought, cognition and perception.

There are several possible explanations for the fact that nurses experience low influences on these patient problems. It may be that nurses simply have low influence on the prevention or minimisation of those types of problems. It could be argued that nurses collaborate with other professionals who are more influential due to their knowledge and competence. On the other hand it is conceivable that nurses are not choosing the correct interventions because they lack the experience or knowledge required to tackle those patient problems. Another explanation is that the patient problems reported are sector‐specific and as such occur more often in a particular sector. Further research should be undertaken to explore why nurses feel this way.

Although the focus of nursing care might differ between clinical settings, our study provides more insights into which patient problems are relevant to clinical nursing practice across different healthcare settings. The problems or health issues that patients experience are not restricted to one specific setting. When a patient with a problem related to attention or memory functions is being transferred from one care setting to another, it is important to exchange the right information to continue appropriate nursing care.

A salient point in this respect is that we are looking at the influence nurses feel they have, not their *actual* influence. While we have no reason to assume that there is a significant difference between the two notions, we have noticed that the above‐mentioned studies investigating patient problems used different vocabularies and classifications. Not only are different terms applied, but the level of detail differs from very specific to more general as well. Moreover, different terms and definitions will lead to inconsistency in outcomes, which will be ineffective in terms of influencing health policy (Hovenga, Garde, & Heard, 2005; Lundberg et al., 2008; Swan, Lang, & McGinley, 2004). The development of unambiguously defined nursing patient problems is an important issue for future research. To ensure that information will be transferred accurately from one healthcare context to another, nurses need to establish a standardised core set of patient problems (Matney et al., 2012), where each patient problem should have a unique term representing its meaning. Although nurses do not perceive a significant influence on the development of relevant nursing information (Gephart, Carrington, & Finley, 2015), they should explore whether a consensus can be reached regarding the various patient problems.

### 4.1 Research strengths and limitations

One positive aspect of this research is that the respondents represent the entire nursing profession – all healthcare sectors are included. A response rate of 52% is acceptable compared with a mean response rate for online surveys of 36.83% (Sheehan, 2001). However, there are limitations to this study. First, nurses in the hospital sector are the largest group of respondents. Second, the mean age of the nurses who participated in our study (49) is higher than the national mean age of nurses working in the healthcare sectors (43) (www.azwinfo.nl; 2014). In addition, 377 respondents (86%) were female, which is somewhat higher than the national proportion of 84% (www.azwinfo.nl). This may affect the extent to which the results can be generalized; the results of our study are however consistent with those of the studies mentioned previously (Doran, 2011; Escalada‐Hernández et al., 2015; MacNeela et al., 2010; Paans & Müller‐Staub, 2015). We have therefore gained more understanding about patient problems that are common in nursing practice and the content underlying them.

Finally, we used medians to create the quadrants to ensure even distributions of the observations. The median for influence divided the problems into problems with less than a moderate level of influence and problems with at least a moderate level of influence. Despite the arbitrary nature of the dividing lines, we gained a better picture of which patient problems are relevant and useful to clinical nursing practice.

## 5 CONCLUSION

The purpose of the current study was to determine which patient problems nurses encounter daily and the nurses’ perceived degree of influence in preventing and minimizing these patient problems. This study found in general that patient problems related to self‐care, such as washing yourself, dressing, toileting and pain occur frequently and that nurses perceive a high level of influence. On the other hand, nurses felt they had less influence on patient problems related to voice/speech or the tasks and actions required to participate in work/employment. The findings of this study enhance our understanding of the patient problems that reflect clinical nursing practice and complement those of earlier studies investigating patient problems. Despite its exploratory nature, the patient problems identified could be used as the foundation for establishing a standardized core set of patient problems to exchange and reuse information within and across different healthcare settings. Overall, this research has increased our knowledge of and insight into patient problems that encapsulate the scope of nursing care.

### 5.1 Implications for nursing practice

This research has revealed an overview of patient problems that encapsulate nursing practice. This finding has important implications for research to find a semantically consistent way of defining patient problems, as is required to exchange or reuse information within and across settings. Besides, nurses and nursing informatics should take the lead in exploring how various patient problems can be described and reported in a consistent manner (unambiguously). Only then will nurses be able to communicate, study the effectiveness of their actions and their contribution to the quality of care provided. Finally, nursing management and policymakers should address the findings of this study. It may provide support for developing and implementing policy to improve the consistency of nursing information capturing nursing practice in electronic health records.

## CONFLICTS OF INTEREST

No conflict of interest has been declared by the authors.

## Appendix 1 Overview of ICF categories and subcategories used in the questionnaire. The definitions are online available at: http://www.who.int/classifications/icf/icfchecklist.pdf?ua=1

### 1 .1

**Table:**

**Category**	**(Problem with) Subcategory**
**b1.**	**MENTAL FUNCTIONS**
1	b110 Consciousness
1	b114 Orientation
1	b117 Intellectual functions
1	b134 Sleep
1	b126 Temperament and personality functions
1	b130 Energy and drive functions
1	b140 Attention functions
1	b144 Memory
1	b152 Emotional functions
1	b156 Perceptual functions
1	b160 Thought functions
1	b180 Experience of self and time functions
**b2.**	**SENSORY FUNCTIONS AND PAIN**
2	b210 Seeing
2	b230 Hearing
2	b250 Taste function
2	b280 Pain and sensation of pain
**b3.**	**VOICE AND SPEECH FUNCTIONS**
3	b310 Voice function
3	b320 Articulation
3	b330 Fluency and rhythm of speech functions
**b4.**	**FUNCTIONS OF THE CARDIOVASCULAR, HAEMATOLOGICAL,IMMUNOLOGICAL AND RESPIRATORY SYSTEMS**
4	b410 Heart functions, including heart rate, rhythm
4	b415 Blood vessel function
4	b420 Blood pressure functions
4	b430 Functions of the haematological system
4	b435 Functions of the immunological system
4	b440 Respiratory system
4	b460 Sensations associated with cardiovascular functions
**b5.**	**FUNCTIONS OF THE DIGESTIVE, METABOLIC AND ENDOCRINE SYSTEMS**
5	b510 Ingestion functions
5	b515 Digestive functions
5	b525 Defecation
5	b530 Weight maintenance
5	b535 Sensations associated with the digestive system
5	b540 Functions related to metabolism system
5	b545 Water, mineral and electrolyte balance functions
5	b550 Thermoregulatory functions
5	b555 Endocrine gland functions
**b6.**	**GENITOURINARY AND REPRODUCTIVE FUNCTIONS**
6	b610 Urinary excretory functions
6	b620 Urination functions
6	b630 Sensations associated with urinary functions
6	b640 Sexual functions
6	b650 Menstruation functions
6	b660 Procreation functions
6	b670 Sensations associated with genital and reproductive functions
**b7.**	**NEUROMUSCULOSKELETAL AND MOVEMENT RELATED FUNCTIONS**
7	b710 Functions of the joints and bones
7	b730 Muscle power functions
7	b735 Muscle tone functions
7	b740 Muscle endurance functions
7	b765 Involuntary movement functions
7	b780 Sensations related to muscles and movement functions
**b8.**	**FUNCTIONS OF THE SKIN AND RELATED STRUCTURESANY OTHER BODY FUNCTIONS**
8	b810 Protective functions of the skin
8	b820 Repair functions of the skin
8	b840 Sensation related to the skin
8	b850‐860 Functions of the hair and nails
**d1.**	**LEARNING AND APPLYING KNOWLEDGE**
9	d110 Sensory experiences
9	d130‐d160 Basic learning and applying knowledge
9	d175 Solving problems
**d2.**	**GENERAL TASKS AND DEMANDS**
10	d210‐d220 Undertaking a single or multiple tasks
10	d230 Carrying out daily routine
10	d240 Handling stress and other psychological demands
**d3.**	**COMMUNICATION**
11	d310‐d325 Communicating ‐ receiving
11	d330‐345 Communicating ‐ producing
11	d350 Conversation
11	d360 Communication devices and techniques
**d4.**	**MOBILITY**
12	d410‐d425 Changing and maintaining body position
12	d430‐d445 Carrying, moving and handling objects
12	d450‐d465Walking and moving
12	d470‐d475 Moving around using transportation
**d5.**	**SELF CARE**
13	d510 Washing oneself
13	d520 Caring for body parts
13	d530 Toileting
13	d540 Dressing
13	d550‐d560 Eating and drinking
13	d570 Looking after one's health
**d6.**	**DOMESTIC LIFE**
14	d610 Acquiring a place to live
14	d620 Shopping and gathering daily necessities
14	d630‐d640 Household tasks
**d7.**	**INTERPERSONAL INTERACTIONS AND RELATIONSHIPS**
15	d710 Basic interpersonal interactions
15	d720 Complex interpersonal interactions, such as forming or terminating relationships
15	d730‐d770 Particular interpersonal interactions, such as relating with strangers, formal relationships, family and intimate relationships
**d8.**	**MAJOR LIFE AREAS**
16	d810‐d830 Education
16	d840‐d855 Work and employment
16	d860‐d870 Economic life
**d9.**	**COMMUNITY, SOCIAL AND CIVIC LIFE**
17	d910 Community life
17	d920 Recreation and leisure
17	d930 Religion and spirituality
